# Nomogram to predict successful smoking cessation in a Chinese outpatient population

**DOI:** 10.18332/tid/127736

**Published:** 2020-10-16

**Authors:** Ning Zhu, Shanhong Lin, Chao Cao, Ning Xu, Xiaopin Yu, Xueqin Chen

**Affiliations:** 1 Department of Respiratory and Critical Care Medicine, Ningbo First Hospital, Ningbo, China; 2 Department of Ultrasound, Ningbo First Hospital, Ningbo, China; 3 Department of Prevention and Health Care, Ningbo First Hospital, Ningbo, China; 4 Department of Traditional Medicine, Ningbo First Hospital, Ningbo, China

**Keywords:** smoking, smoking cessation, predictors, nomogram

## Abstract

**INTRODUCTION:**

The study aimed to establish and internally validate a nomogram to predict successful smoking cessation in a Chinese outpatient population.

**METHODS:**

A total of 278 participants were included, and data were collected from March 2016 to December 2018. Predictors for successful smoking cessation were evaluated by 3-month sustained abstinence rates. Least absolute shrinkage and selection operator (LASSO) regression was used to select variables for the model to predict successful smoking cessation, and multivariable logistic regression analysis was performed to establish a novel predictive model. The discriminatory ability, calibration, and clinical usefulness of the nomogram were determined by the concordance index (C-index), calibration plot, and decision curve analysis, respectively. Internal validation with bootstrapping was performed.

**RESULTS:**

The nomogram included living with a smoker or experiencing workplace smoking, number of outpatient department visits, reason for quitting tobacco, and varenicline use. The nomogram demonstrated valuable predictive performance, with a C-index of 0.816 and good calibration. A high C-index of 0.804 was reached with interval validation. Decision curve analysis revealed that the nomogram for predicting successful smoking cessation was clinically significant when intervention was conducted at a successful cessation of smoking possibility threshold of 19%.

**CONCLUSIONS:**

This novel nomogram for successful smoking cessation can be conveniently used to predict successful cessation of smoking in outpatients.

## INTRODUCTION

According to the 2017 World Health Organization report^1-5^, tobacco smoking remains a major worldwide public health threat, with >7 million deaths directly related to tobacco. Many studies have recognized smoking as a risk factor for chronic diseases, such as chronic respiratory diseases (asthma, chronic obstructive pulmonary disease), hypertension, cardiovascular disease, atherosclerosis, diabetes, cancer, and microbial infections (respiratory infections, bacterial meningitis)^6,7^. Smoking also burdens healthcare systems and increases social costs. Given the impact of cigarette smoking, the development of effective interventions to address tobacco addiction is a major public health need. There are 350 million smokers in China, which accounts for one-third of the world’s smokers^8^. Unfortunately, smoking cessation services and counseling are at an early stage of development in China. Moreover, healthcare workers in China do not exert much effort in helping smokers to quit tobacco use^9^. Therefore, the effectiveness of existing smoking cessation interventions and services are largely unknown in China.

Numerous prior studies have examined individuallevel predictors of successful and unsuccessful cessation attempts^2,10,11^, including socio-economic status groups, increasing tobacco prices, both peer and family smoking groups, and use of smoking cessation medications, but researchers have come to differing conclusions. In addition, another study shows that it is important to understand the differential roles that pre-quitting and post-quitting experiences play in smoking cessation and to provide help to smokers for not resuming cigarette smoking^12^. It is important to understand the characteristics of smokers and identify factors predicting successful smoking cessation to improve the efficacy of interventions. Some smokers are unwilling (i.e. not ready, not motivated, or not able) to attempt quitting in the near future, so identification of predictors and determinants of success in smoking cessation is a key component in smoking cessation programs. Therefore, we assessed the outcomes of smokers in our smoking cessation clinic and investigated factors predictive of successful smoking cessation treatment. The primary objective of this study was to develop a valid but simple prediction tool by using only characteristics easily determined at the beginning of treatment to assess the factors associated with successful smoking cessation, with the goal of enabling physicians in smoking cessation clinics to provide individualized treatment strategies.

## METHODS

### Study participants

A total of 278 smokers treated at the smoking cessation clinic of Ningbo First Hospital from March 2016 to December 2018 were enrolled in the study. The inclusion criteria were current smokers (smoked daily for ≥12 months at the time of the survey), aged ≥18 years, motivated to quit, and willing to participate in the follow-up visits. Additionally, smokers whose intention to quit was not clear at the first visit but who had a desire to quit after smoking cessation counseling were also included in the study group. The exclusion criteria were smokers who did not want to participate in a cessation program even after smoking cessation counseling and smokers who were unwilling or unable to receive regular follow-up. The present study was performed with the informed consent of each subject and with the approval of the local Ethics Committee of Ningbo First Hospital (Ningbo, China).

### Data collection

The smoking cessation clinic physician completed baseline data collection at the first visit. All participants filled in questionnaires addressing treatment-related information and participated in face-to-face interviews with the physicians of the smoking cessation clinic. Before counseling, the smokers filled in a questionnaire addressing the following information: age, gender, marital status, educational level, employment, working strength, alcohol usage, monthly per capita income (RMB: 1000 Renminbi about 150 US$), and comorbidities. Additionally, questions about tobacco-related factors and treatment characteristics, including average number of cigarettes smoked daily, age when started smoking, smoking duration, average daily cost of cigarettes, living with a smoker or experiencing workplace smoking, prior attempts to quit smoking, reason for quitting smoking, current smoking status (smoking or quit), time after waking up to smoking the first cigarette (min), number of outpatient department visits, and use of varenicline or bupropion, were assessed. Exhaled carbon monoxide (CO, parts per million, ppm) levels were measured by trained technicians using a standard with a Micro Smokerlyser. Nicotine dependency was calculated according to the Fagerström Test for Nicotine Dependence (FTND) score^13^. The degree of nicotine dependency was graded by using the FTND score, which consisted of six questions: number of cigarettes smoked per day (≤10 = 0; 11–20 = 1; 21–30 = 2; ≥31 = 3); time to first cigarette of the day (>60 minutes = 0; 31–60 minutes = 1; 6–30 minutes = 2; 0–5 minutes = 3); difficulty not smoking in no-smoking areas (no = 0; yes = 1); which cigarette would the smoker hate most to give up (first of the morning = 1; others = 0); smoke more frequently in first hours after waking (no = 0; yes = 1); and smoke when ill in bed (no = 0; yes = 1). The degree of nicotine dependency was graded by using the FTND score, with suggested thresholds for mild (0–3), moderate (4–6), and severe (7–10).

We required the participants to receive the standard-dose therapy of varenicline or bupropion following the manufacturers’ prescribing information for 4 weeks prior to a target quit date (TQD), and to receive varenicline or bupropion therapy for 12 weeks in total, while also being introduced to smoking cessation techniques and undergoing psychological counseling.

### Follow-up at later stage

The participants were followed up for 6 months after the baseline visit, including outpatient followup, telephone, or Wechat follow-up (WeChat is the most commonly used and popular social networking software in China; almost every adult has a WeChat number on their mobile phone). At least one followup visit to the smoking cessation clinic prior to TQD was required. During the Wechat follow-up, the attending physician sent an invitation code at the first visit and joined the Wechat quitting group after being confirmed by the smokers. The smoking cessation knowledge content was very comprehensive and included texts, pictures, psycho-educational audios and videos, recording of smoking history, and progress visualized through graphics, which was sent to smokers twice a week through the WeChat group in the first 4 weeks and once a week from weeks 5 to 24. Smokers could communicate through the Wechat platform at any time if they encountered problems in the process and receive detailed answers from a physician.

The primary outcome was evaluated by the 3-month continuous cessation rate, which was defined as self-reported quitting ≥3 months at the follow-up at 6 months, with verification by a measured exhaled carbon monoxide (CO) level of ≤6 ppm.

### Statistical analysis

Characteristics associated with smoking cessation selected by the least absolute shrinkage and selection operator (LASSO) regression model were used in multivariate logistic regression analysis to establish a smoking cessation prediction model. All statistical analyses were performed using R software (version 3.6.5; the R Foundation, https://www.r-project.org/). Based on the primary data set, the LASSO method suitable for high-dimension data reduction^14,15^ was then used to reduce the data dimensions and identify the optimal cessation predictors for modeling. Features with non-zero coefficients in the LASSO regression model were selected^16^. These predictors were used in multivariable analysis to establish the predictive model. Multivariate binary logistic regression was performed with the ‘rms’ package. The nomogram was constructed, and calibration curve plots were generated by using the ‘rms’ package. Decision curve analysis (DCA) was performed by using DCA.R. A bootstrap calculation of 1000 resamples was performed on the nomogram to calculate the C-index to demonstrate the discrimination capacity of the nomogram. A calibration chart was used to prove the consistency, and DCA was used to evaluate the net benefit of the nomogram. The reported levels of statistical significance were two-tailed, with statistical significance accepted for p<0.05.

## RESULTS

### Patient characteristics

A total of 298 smokers were recruited for the study. During the follow-up, 20 smokers dropped out of the study because they could not complete the follow-up or did not receive standard medication as scheduled. In total, 278 smokers completed the smoking cessation program and were divided into the smoking quitter and non-quitter groups for the later analysis. The smokers in the quitter group met the 3-month continuous cessation rate criteria, and all others were assigned to the non-quitter group. In addition, only 14.5% (37/263) of the drug-assisted quitters withdrew from the drug treatment due to economic reasons or failure to standardize their use of the drug, and the others adhered to the drug treatment instructions for 12 weeks, which was also the duration of the medication course specified in the drug instructions. All patient data, including basic demographic characteristics, tobacco-related factors and treatment features are shown in Table 1.

**Table 1:** Differences in demographic and clinical characteristics between smoking quitters and nonquitters

*Characteristics*	*Non-Quitters (n=113) n (%)*	*Quitters (n=165) n (%)*	*Total (n=278) n (%)*
**Age** (years)			
<30	14 (12.4)	14 (8.5)	28 (10.1)
30–50	70 (61.9)	104 (63.0)	174 (62.6)
>50	29 (25.7)	47 (28.5)	76 (27.3)
**Gender**			
Female	2 (1.8)	7 (4.2)	9 (3.2)
Male	111 (98.2)	158 (95.8)	269 (96.8)
**Marital status**			
Married	88 (77.9)	132 (80.0)	220 (79.1)
Other	25 (22.1)	33 (20.0)	58 (20.9)
**Educational level**			
Primary (0–9 years)	47 (41.6)	56 (33.9)	103 (37.1)
Secondary (9–12 years)	26 (23.0)	52 (31.5)	78 (28.1)
Higher (>12 years)	40 (35.4)	57 (34.5)	97 (34.9)
**Employment**			
Student/unemployed/retired/other	27 (23.9)	41 (24.8)	68 (24.5)
Currently employed	86 (76.1)	124 (75.1)	210 (75.5)
**Working strength**			
Low activity (office, etc.)	35 (31.0)	66 (40.0)	101 (36.3)
Light-to-moderate activity			
(installers, etc.)	44 (38.9)	52 (31.5)	96 (34.5)
Moderate or heavy activity (agriculture, etc.)	34 (30.1)	47 (28.5)	81 (29.1)
**Alcohol consumption**			
No	39 (34.5)	51 (30.9)	90 (32.4)
Yes	74 (65.5)	114 (69.1)	188 (67.6)
**Monthly income** (RMB)^a^			
<5000	41 (36.3)	54 (32.7)	95 (34.2)
5000–10000	43 (38.0)	61 (37.0)	104 (37.4)
>10000	29 (25.7)	50 (30.3)	79 (28.4)
**Comorbidities**			
None	53 (46.9)	62 (37.6)	115 (41.4)
Respiratory	21 (18.6)	77 (46.7)	98 (35.2)
Bronchitis	13 (11.5)	50 (30.3)	63 (22.7)
Asthma	3 (2.7)	4 (2.4)	7 (2.5)
Lung cancer	2 (1.8)	9 (5.5)	11 (1.0)
Other	3 (2.7)	14 (8.5)	17 (6.1)
Non-respiratory	39 (34.5)	26 (15.7)	65 (23.4)
Diabetes	5 (4.4)	6 (3.6)	11 (1.0)
Cardiovascular disease	12 (10.6)	9 (5.5)	21 (7.6)
Hypertension	4 (3.5)	2 (1.2)	6 (2.2)
Hyperlipidemia	3 (2.7)	1 (0.1)	4 (1.4)
Other	15 (13.3)	8 (4.8)	23 (8.3)
**Cigarettes smoked on average daily**			
<10	27 (23.9)	30 (18.2)	57 (20.5)
10–20	52 (46.0)	69 (41.8)	121 (43.5)
>20	34 (30.1)	66 (40.0)	100 (36.0)
**Age started smoking** (years)			
<18	29 (25.7)	47 (28.5)	76 (27.3)
≥18	84 (74.3)	118 (71.5)	202 (72.7)
**Smoking duration** (years)			
<10	58 (51.3)	74 (44.8)	132 (47.5)
10–20	37 (32.7)	57 (34.5)	94 (33.8)
>20	18 (15.9)	34 (20.6)	52 (18.7)
**Average daily cost of cigarettes** (RMB)			
<40	27 (23.9)	42 (25.5)	69 (24.8)
≥40	86 (76.1)	123 (74.5)	209 (75.1)
**Living with a smoker or experiencing workplace smoking**			
No	22 (19.5)	98 (59.4)	120 (43.2)
Yes	91 (80.5)	67 (40.6)	158 (56.8)
**Prior attempts to quit smoking**			
0	40 (35.4)	62 (37.6)	102 (36.7)
≥1	73 (64.6)	103 (62.4)	176 (63.3)
**Reason for quitting smoking**			
Prevention and treatment of own diseases	17 (15.0)	101 (61.2)	118 (42.4)
Mobilization of others	52 (46.0)	38 (23.0)	90 (32.4)
Others	44 (38.9)	26 (15.8)	70 (25.2)
**Quit smoking status**			
Not decided	42 (37.2)	60 (36.4)	102 (36.7)
Intent	48 (42.5)	64 (38.8)	112 (40.3)
Already started	23 (20.4)	41 (24.8)	64 (23.0)
**The time to first cigarette on waking** (minutes)			
<5	38 (33.6)	63 (38.2)	101 (36.3)
6–60	52 (46.0)	63 (38.2)	115 (41.4)
>60	23 (20.4)	39 (23.6)	62 (22.3)
**FTND score**			
Low (0–3)	10 (8.8)	14 (8.5)	24 (8.6)
Moderate (4–6)	46 (40.7)	75 (45.5)	121 (43.5)
Severe (7–10)	57 (50.4)	76 (46.0)	133 (47.8)
**Exhaled carbon monoxide** (ppm)			
0–6	17 (15.0)	18 (10.9)	35 (12.6)
7–10	36 (41.9)	42 (25.5)	78 (28.1)
11–20	50 (44.2)	96 (58.2)	146 (52.5)
>20	10 (8.8)	9 (5.5)	19 (6.8)
**Outpatient department visits**			
1	83 (73.5)	88 (53.3)	171 (61.5)
≥2	30 (26.5)	77 (46.7)	107 (38.5)
**Varenicline use**			
No	18 (15.9)	14 (8.5)	32 (11.5)
Yes	95 (84.1)	151 (91.5)	246 (88.5)
**Bupropion use**			
No	105 (92.9)	156 (94.5)	261 (93.9)
Yes	8 (7.0)	9 (5.5)	17 (6.1)

a RMB: 1000 Chinese Renminbi about 150 US$.

### Feature selection

**Figure 1:**
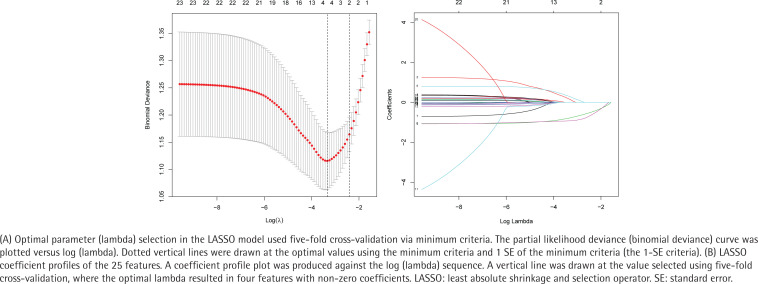
Demographic and clinical feature selection using the LASSO binary logistic regression model

Based on 278 patients in the cohort, 25 features were reduced to four potential predictors (Figures 1A and B), and the coefficients were non-zero in the LASSO regression model: reason for quitting smoking, number of other smokers in the household, number of visits to the outpatient department, and varenicline use (Table 2).

**Table 2:** Factors predictive of successful smoking cessation

	*β*	*OR (95% CI)*	*p*
**Intercept**	1.2693	3.558 (1.164–11.132)	0.027
**Reason for quitting smoking**			
Mobilization of others vs prevention and treatment of own diseases	−2.1006	0.122 (0.056–0.255)	<0.001
Others vs prevention and treatment of own diseases	−2.2011	0.111 (0.049–0.239)	<0.001
**Living with a smoker or being exposed to workplace smoking** yes vs no	−0.8220	0.439 (0.219–0.878)	0.020
**Number of outpatient department visits** ≥2 vs 1	1.0526	2.865 (1.439–5.844)	0.003
**Varenicline use** yes vs no	0.6943	2.002 (0.825–4.999)	0.128

OR: odds ratio. β: regression coefficient.

### Generation of an individualized prediction model

**Figure 2:**
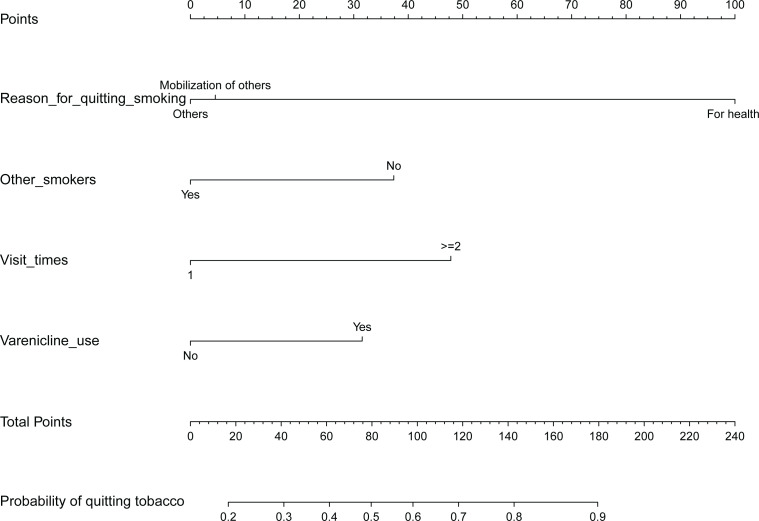
Nomogram to predict the probability of quitting smoking

Based on the multivariate analysis results, predictive factors, including the reason for quitting smoking, living with a smoker or being exposed to workplace smoking, number of visits to the outpatient department, and varenicline use, were incorporated into the nomogram; these characteristics are shown in Table 2. A model containing the above independent predictors was established and is represented as a nomogram in Figure 2.

### Apparent performance of the nomogram for prediction of successful smoking cessation

**Figure 3:**
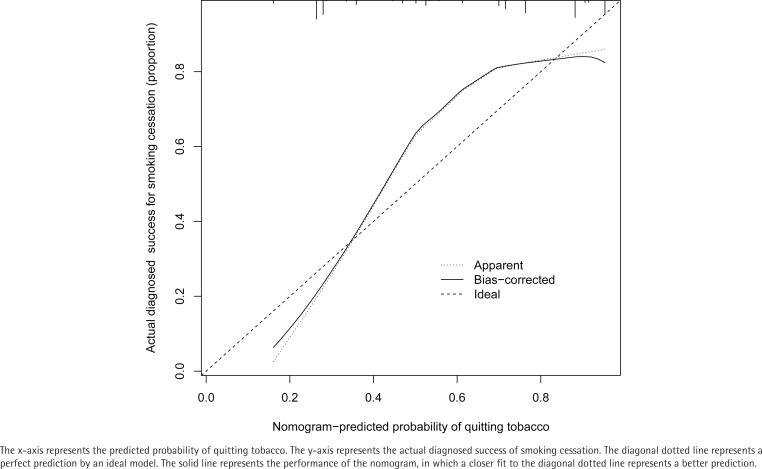
Calibration curves of nomogram prediction of successful smoking cessation in the cohort

The calibration curve of the nomogram for prediction of successful smoking cessation showed good consistency (Figure 3). The C-index of the predictive nomogram was 0.816 (95% CI: 0.761– 0.871) for this cohort and was confirmed by internal validation as 0.804, indicating that this model had good discriminatory ability. The nomogram had good power for predicting success in quitting smoking.

### Clinical value of the model

**Figure 4:**
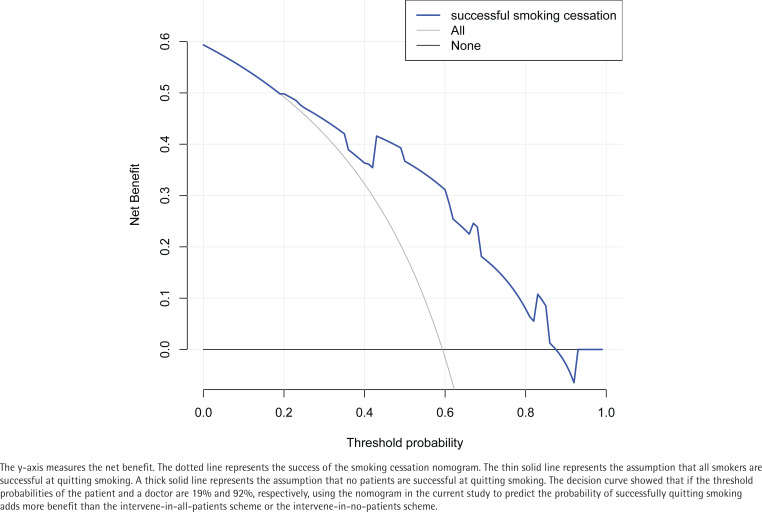
Decision curve analysis for the nomogram predicting successful smoking cessation

The DCA for predicting the success of quitting smoking is shown in Figure 4. The decision curve shows that the use of this nomogram increased the ability to predict successful smoking cessation when the patient and physician threshold probabilities were 19% and 92%, respectively. In this range, according to the successful smoking cessation nomogram, the net benefit was comparable to several overlaps.

## DISCUSSION

In the analysis of predictors of quitting smoking and reasons for quitting, living with a smoker or being exposed to workplace smoking, number of outpatient department visits, and varenicline use were associated with successful cessation rate. This nomogram suggested that treatment with varenicline, quitting for health-related reasons, more visits, and not coexisting with other smokers may be key factors that determine the success of smoking cessation.

The efficacy data in our study showed that varenicline was more effective than bupropion for smoking cessation. However, there is strong evidence from multiple randomized clinical trials that both bupropion and varenicline increased smoking cessation rates when used in a quitting attempt^17,18^. One potential explanation of this discrepancy between our results and those of the trials is that the low-usage rate of bupropion may have resulted in underestimation of its potential efficacy.

To our surprise, the number of outpatient department visits was the most influential factor affecting smoking cessation. In addition, we found that the reason for quitting smoking could predict the success of the attempt. These results indicate that individual motivation, especially intrinsic motivation, was predictive of the smoking cessation result. These results are in accordance with those of many reports from the medical literature^12,19-21^, which suggest that smokers subjectively recognize the harm of smoking and the benefits of quitting smoking and that taking the initiative to quit smoking is very important for success. From a pooled estimate of 65 trials, Hartmann–Boyce et al.^22^ concluded that increasing the amount of behavioral support is likely to increase the chance of success by approximately 10% to 20%. Smokers with more outpatient visits may be able to obtain more behavioral and psychosocial support, thus achieving better smoking cessation results.

As the results show, not co-existing with other smokers also was a predictor of smoking cessation success. This finding is consistent with the results of previous reports that living with a smoker or being exposed to workplace smoking made individuals less likely to quit^23-26^. It is possible that exposure to other people smoking decreases quitting rates and increases the risk of starting to smoke^23^. Smoking is not only a personal behavior in China, which has a high smoking rate, but also deeply influenced by social factors. Smokers who are often surrounded by other smokers perceive higher approval and acceptance of smoking behavior, thus further strengthening smoking behavior. From this point of view, we should pay more attention to decreasing passive smoking. It should be noted that age, sex, education, occupation, and health status were not predictive factors for the success of smoking cessation in our study. Although the investigators observed that some of these factors were independent predictors^21,27,28^, the findings of our study appeared to be inconsistent with some published evidence and could not confirm all the previous findings. This inconsistency may be because of differences between countries and regions. Some studies have found that measuring exhaled CO levels were a useful biomarker for predicting successful smoking cessation^3-5^. However, in our study, exhaled CO levels at the first visit were not associated with success in quitting. In addition, neither the average number of cigarettes smoked daily nor the FTCD scores related to nicotine dependence were associated with the success of smoking cessation. This finding differs from the results in previous studies^19,^ ^29,30^. In their study, Huang et al.^5^ found that smokers with lower FTCD scores, those with lower exhaled CO concentrations, and those who smoked <20 cigarettes per day on average, had higher success rates. These differences in conclusions may be because our follow-up time was limited, the self-reports may have underestimated cigarette consumption, and only the first measurements of CO levels were compared.

In our study, the 3-month continuous abstinence rate of 59.4% (165/278) was indeed a relatively high level, compared with other studies that generally report proportions of quitting lower than 50%. We think that it may be related to the following reasons. First, because most Chinese smokers think drug therapy is the most important type of treatment, psychological therapy and behavioral therapy could only play a supplementary role. Drug therapy is the beginning of medical behavior for many patients. We have tried to correct smokers’ perceptions many times, but the effect of our previous efforts was not obvious. In our study, 94.6% (263/278) of the enrolled smokers used adjuvant treatment with smoking cessation drugs, especially varenicline, and received a standard course of treatment. Studies have proved that smoking cessation drugs, especially varenicline, obviously improved the success rate of smoking cessation. Second, the choice of medications and timing of giving smoking cessation drugs were also important factors. Varenicline has an acceptable safety/tolerability profile and has been proven to be more effective than placebo, bupropion, and NRT, in the general population^31-33^. In a flexible quit date study that evaluated this paradigm (n=659), continuous abstinence rates of weeks 9–12 (CAR9– 12) were significantly higher with varenicline than with placebo (53.1% vs 19.3%, respectively), as were CAR9–24^34^. Hajek et al.^35^ hypothesized that if varenicline reduced the rewarding properties associated with cigarette smoking, pre-loading the drug prior to TQD could help weaken this association and enhance efficacy. In the study, participants (n=101) received varenicline for 4 weeks prior to the TQD and received varenicline for 12 weeks. The study found that the effect on early quitting rates (CARs at week 4) did not reach statistical significance (varenicline 49.1% vs placebo 33.3%), but there was a statistically significant improvement in self-reported abstinence at 12 weeks (varenicline 47.2% vs placebo 20.8%). In our study, pre-loading of the drug prior to TQD also gave similar results. As discussed earlier, studies that further investigate varenicline pre-loading could confirm the potential benefits of this approach. While currently not included in the prescribing information, varenicline pre-loading^35-37^ has shown improved efficacy, particularly in highly dependent smokers. Third, the popularity of smart phones eliminates the limitation of time and space. Popular mobile social software, such as WeChat, shows great potential in promoting healthy behavioral changes in China. We have developed systematic follow-up mechanisms, including sending smokers vivid and easily understood smoking cessation information regularly and extensively. These measures ensure continuous psychological and behavioral support for smokers. In addition, one of the reasons for the high cessation rate is that our doctors patiently provide timely answers and help answer smokers’ questions or clarify points of confusion. Unfortunately, in China, smoking cessation drugs are not included in the scope of medical insurance reimbursement. It is hoped that more work will be done in this area.

### Limitations

Some circumstances may limit the generalizability of our findings. First, the sample size was not sufficiently large; thus, the statistical power was diminished, which may underlie the null-effect findings. Moreover, most participants were male, and the characteristics and predictors of success for smoking cessation in females remain unclear. The cohort was not representative of all Chinese smoking cessation outpatient populations. Second, factors that motivate smokers to attempt quitting are very different from those involved in maintaining abstinence^9^. Because of the relatively short time span of 6 months follow-up, uncertainty remains as to whether changes in behavior can be maintained over a longer time span, making the findings of this study insufficient for predicting long-term smoking cessation outcomes. Third, the predictor analysis did not include all potential factors that affect smoking cessation. Some possible aspects of smoking cessation were not thoroughly investigated, such as depression–anxiety psychological factors and other relevant conditions. Considering the above limitations, we need well-designed large-sample studies with longer follow-up durations to confirm our findings. In addition, although the robustness of our nomogram has been extensively verified by the internal bootstrap test, it could not be verified externally, so generalizability to other smoking cessation populations in other regions and countries is uncertain. This nomogram requires external assessment in a broader population of smokers.

## CONCLUSIONS

We developed a novel nomogram with relatively good accuracy to help clinicians assess the probability of a smoker successfully quitting after initiation of smoking cessation treatment, which is also the question smokers most want to know when they are ready to quit. By evaluating the predictors of success, clinicians can target more important smoking cessation interventions, such as more individualized behavioral therapy, psychotherapy, or medication. This nomogram requires external validation, and further studies are needed to determine if individual interventions based on this nomogram can improve the success rates and effectiveness of smoking cessation therapies.
